# Characterization of Films with Thickness Less than 10 nm by Sensitivity-Enhanced Atomic Force Acoustic Microscopy

**DOI:** 10.1007/s11671-010-9778-8

**Published:** 2010-09-26

**Authors:** Mikio Muraoka, Shinji Komatsu

**Affiliations:** 1 Department of Mechanical Engineering, Akita University, 1-1 Tegatagakuen-machi, Akita 010-8502, Japan

**Keywords:** Atomic force acoustic microscopy, Thin film, Elastic modulus, Diamond-like carbon, Concentrated-mass cantilever

## Abstract

We present a method for characterizing ultrathin films using sensitivity-enhanced atomic force acoustic microscopy, where a concentrated-mass cantilever having a flat tip was used as a sensitive oscillator. Evaluation was aimed at 6-nm-thick and 10-nm-thick diamond-like carbon (DLC) films deposited, using different methods, on a hard disk for the effective Young's modulus defined as *E*/(1 - *ν*^2^), where *E* is the Young's modulus, and *ν* is the Poisson's ratio. The resonant frequency of the cantilever was affected not only by the film's elasticity but also by the substrate even at an indentation depth of about 0.6 nm. The substrate effect was removed by employing a theoretical formula on the indentation of a layered half-space, together with a hard disk without DLC coating. The moduli of the 6-nm-thick and 10-nm-thick DLC films were 392 and 345 GPa, respectively. The error analysis showed the standard deviation less than 5% in the moduli.

## Introduction

The protective coating for hard disks, namely a diamond-like carbon (DLC) film, is now targeted for thickness less than 3 nm because of the reduced spacing between the magnetic layer and the read/write head [1]. The mechanical properties become very important for reliability of the devices. The chemical structure of DLC significantly depends on the deposition process and influences the mechanical properties such as elasticity and hardness. Especially the Young's modulus *E* drastically varies with a content of *sp*^3^-bonds, which form three-dimensional interlinks in the amorphous network of carbons (*E* ≈ 100–800 GPa) [2-4]. Therefore, the modulus is useful to identify the chemical structure of films.

Various approaches for the determination of the elastic properties of thin films have been previously used, including nanoindentation [5], laser spectroscopic methods [3], and removed substrate methods [4]. However, it is still a challenging problem to evaluate ultrathin films like DLC films with thickness less than 10 nm.

Atomic force acoustic microscopy (AFAM) [6] is a promising method, which belongs to a family of dynamic techniques of atomic force microscope (AFM) such as micro-deformation microscopy [7] and ultrasonic atomic force microscopy [8]. AFAM measures the resonant frequency *f* of an AFM cantilever whose sensor tip is in contact with a sample oscillated by a piezoelectric device. If an appropriate order of the vibration mode is selected, *f* varies with the contact stiffness k*, namely the interactive force gradient between a tip and a sample. The effective Young's modulus $E_{\text{s}}^{*}$ of a sample, defined as $E_{\text{s}}/(1-\nu_{\text{s}}^{2})$ (*E*_s_: the Young's modulus, *ν*_s_: the Poisson's ratio), is evaluated using contact mechanics relating k* to $E_{\text{s}}^{*}$.

Characterization of a 50-nm-thick Ni film deposited on a Si substrate was demonstrated in AFAM, where *f* was observed without the substrate effects [9]. In regard to DLC thin films, only relative evaluation was performed [10]. These studies required a blunt tip with a radius of about 200 nm and a stiff cantilever of spring constant *k*_c_ ≈ 50 N/m to realize reproducible measurements. However, the requirement reduced the spatial resolution and the sensitivity in detection of the contact force.

When attempting to analyze difficult samples like a DLC film with thickness less than 10 nm, higher performance of AFAM is required on the detection of k* and the spatial resolution. We previously proposed a concentrated-mass (CM) cantilever as a way of enhancing the sensitivity in k*-detection without trade-offs [11]. A CM cantilever assures the maximum sensitivity for any sample material. Also, a flat tip with ductile-metal coating, keeps a stable contact area of a radius less than 5 nm and drastically simplifies the relation between k* and $E_{\text{s}}^{*}$[12].

The method we previously developed, termed sensitivity-enhanced AFAM [12], is extended in this letter to the determination of the elastic modulus of ultrathin films. The demonstration was carried out for DLC films with thickness of 6 and 10 nm, deposited on a hard disk. A curve relating *f* to $E_{\text{s}}^{*}$ was determined from multiple measurements on reference samples. The uncertainty was discussed by error analysis. In the evaluation of the DLC-coated samples, the substrate effect was taken into account by using an analytical model for indentation of a layered half-space [13].

## Experimental Procedure and Theory

### CM Cantilever and Apparatus

**Figure 1:**
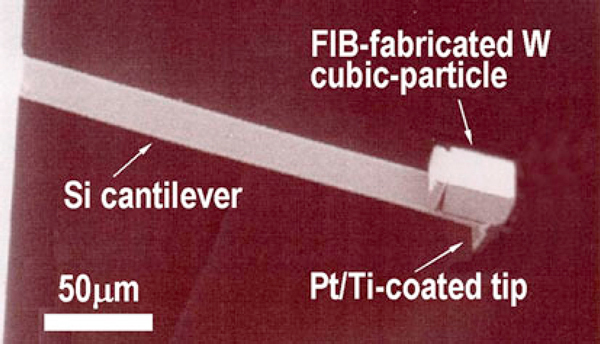
**The concentrated-mass (CM) cantilever. The CM was micro-machined from a 20-μm-thick tungsten film using a focused ion beam (FIB)**.

The experimental procedure is described elsewhere in detail [11,12]. We will briefly explain it here. The main body of a CM cantilever was a rectangular cantilever made of single-crystalline silicon (μMasch Co. Ltd., *k*_c_ = 0.65 N/m, fundamental resonant frequency 40.9 kHz). The silicon tip had an apex radius of about 10 nm and was coated with a 25-nm-thick Pt/Ti film. The coated tip was plastically deformed on a flat diamond surface under a contact load of 2 μN to give it a flat-ended shape. This plastic deformation also induced a work-hardening of the coating, which would prolong the lifetime of the coated tip [12]. For the concentrated mass, a tungsten (W) particle of 35 × 33 × 20 μm in size was micro-machined from a W sheet of 20 μm thick by focused ion beam (FIB). The particle's mass was about 445 ng, which corresponds to a mass ratio of 10.9, namely the ratio of the particle's mass to the silicon-cantilever's mass. The particle was attached adhesively to the free end of the cantilever by micromanipulation. Figure 1 shows a scanning electron micrograph of the CM cantilever. The main difference from the previous works [11,12] was in the use of the micro-machined particle instead of a deoxidized random particle for the concentrated mass. Another difference was in the process that a flat tip was formed from a virgin tip, not from a tip wasted after several tens of scans for imaging.

An atomic force microscope (SII Co. Ltd., SPI3700-SPA270) was used in so-called contact mode for observing the contact resonance spectra. The amplitude of cantilever vibration was acquired with a lock-in amplifier through a heterodyne down-converter. A piezoelectric device placed beneath a sample was used for the oscillation. The time-averaged cantilever deflection signal, which corresponds to the contact force *F*_e_, was maintained through a built-in feedback circuit, where the electronic circuit is not subjected to sinusoidal signals at ultrasonic frequencies. The resonant frequency was measured at five to ten different locations on a sample to confirm reproducibility. All experiments were carried out at a temperature of 20–25°C and relative humidity of about 40–50%.

### Reference and DLC Samples

The reference samples and the elastic moduli are listed in Table 1. We employed a sapphire (0001) wafer in addition to silicon wafers and a diamond (100) used in the previous work [12]. These values were deduced from the crystal moduli determined by ultrasonic velocity techniques for bulk samples (see appendix for sapphire).

**Table 1:** Elastic moduli of reference samples

Reference sample	**Young's modulus *E***_**s **_**(GPa)**	**Poisson's ratio (*ν***_**s**_**)**	**Effective Young's modulus **$E_{\text{s}}^{*}=E_{\text{s}}/(1-\nu_{\text{s}}^{2})$**[GPa]**
Diamond (100)	1050	0.1	1061
Sapphire (0001)	451.2	0.172	465.1
Silicon (111)	187.9	0.180	194.2
Silicon (100)	130.0	0.278	140.9

DLC films of 6 nm thick and 10 nm thick were deposited on a substrate by sputtering a carbon target in Ar gas and by plasma-assisted chemical vapor deposition (CVD), respectively. The film thickness was estimated based on the deposition time. The substrate was a hard disk, which consisted of metallic multi-layers for magnetic record and a glass substrate, namely (50-nm-thick Co-Cr-alloy layer)/(70-nm-thick Ti-alloy layer)/(0.6-mm-thick glass substrate). Also, the substrate without DLC coating was tested for the elastic modulus.

### Theory for Evaluation of Thin Films

The resonant frequency (*f*) of a CM cantilever increases with the contact stiffness (k*) in accordance with the spring-mass model, namely k*/*k*_c_ = (*f*/*f*_0_)^2^, where *f*_0_ is the fundamental resonant frequency in the absence of a sample. A flat tip maintains a constant contact area independent of the adhesion force and the contact force. This also ensures constant k*. The theoretical formula k* = 2aE* for a flat-ended punch [14] is applicable, where *a* is the radius of the contact area. E* is the effective Young's modulus of the contact region, defined as $1/E^{*}=1/E_{\text{t}}^{*}+1/E_{\text{s}}^{*}$. $E_{\text{t}}^{*}[=E_{\text{t}}/(1-\nu_{\text{t}}^{2})]$ is the effective Young's modulus of a tip. These equations give the formula relating *f* to $E_{\text{s}}^{*}$[12]:

(1) $$f=\sqrt{\frac{2AE_{\text{t}}^{*}E_{\text{s}}^{*}}{E_{\text{t}}^{*}+E_{\text{s}}^{*}}},$$

where $A(=a\;f_{0}^{2}/k_{\text{c}})$ is a factor proportional to the contact radius. Both $E_{\text{t}}^{*}$ and *A* can be determined from the *f* measurements for reference samples.

**Figure 2:**
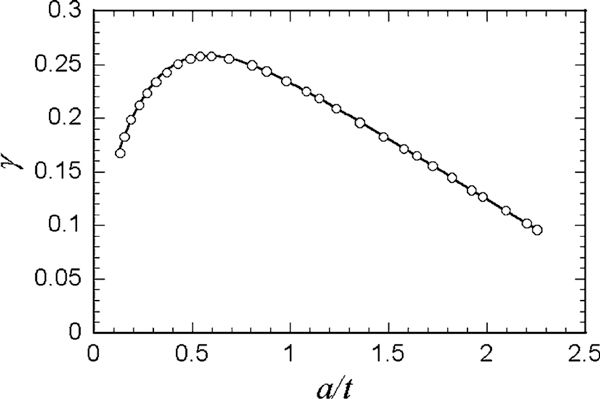
**Relationship between *γ* and *a*/*t*, where the symbol ο represents the numerical result obtained by the theoretical analysis for indentation of a layered half-space **[13]**, and the solid curve is a least-square fit of Eq. 3**.

Analytical models on indentation of a layered half-space for a circular punch proved the validity of the following empirical formula [13]:

(2) $$\frac{1}{E_{\text{s}}^{*}}=\frac{1}{E_{\text{film}}^{*}}\left[1-\exp\left(-\frac{\gamma t}{a}\right)\right]+\frac{1}{E_{\text{sub}}^{*}}\exp\left(-\frac{\gamma t}{a}\right),$$

where $E_{\text{film}}^{*}$ and $E_{\text{sub}}^{*}$ are the effective Young's moduli of a film and a substrate, respectively. The coefficient *γ* is a function of *a*/*t*, where *t* is the film thickness. The numerical result on a relation of *γ* and *a*/*t* was graphically shown in reference [13]. Note that the symbol *a* in reference [13] is defined as the square root of the contact area, which differs from the definition of *a* (the radius of the contact area) in this letter, and then *γ* multiplied by π^1/2^ equals the symbol *α* in reference [13]. Examples of the numerical result are indicated with circles in Figure 2. The numerical data can be well fitted by the following formula.

(3) $$\gamma =\frac{c_{0}{\left(a/t\right)}^{n0}}{{\left(a/t\right)}^{n1}+c_{2}{\left(a/t\right)}^{n2}+c_{3}},$$

where *c*_0_ = 0.4684, *c*_2_ = 0.009968, *c*_3_ = 1.004, *n*0 = 0.4910, *n*1 = 1.736, and *n*2 = 6.607 are the coefficients determined by a nonlinear least-square fit.

Errors in $E_{\text{s}}^{*}$ for a sample come from uncertainties in the predetermination of *A* and $E_{\text{t}}^{*}$ and in the *f* measurement for the sample, which are represented by the standard deviations *σ*_A_, *σ*_Et_, and *σ*_f_, respectively. The standard deviation *σ*_Es_ of $E_{\text{s}}^{*}$ can be estimated by the error propagation on Eq. 1: $\sigma_{\text{Es}}^{2}=D_{\text{A}}^{2}\sigma_{\text{A}}^{2}-2D_{\text{A}}D_{\text{Et}}\sigma_{\text{A}}\sigma_{\text{Et}}+D_{\text{Et}}^{2}\sigma_{\text{Et}}^{2}+D_{\text{f}}^{2}\sigma_{\text{f}}^{2}$, where $D_{\text{A}}=\partial E_{\text{s}}^{*}/\partial A,D_{\text{Et}}=\partial E_{\text{s}}^{*}/\partial E_{\text{t}}^{*}$ and $D_{\text{f}}=\partial E_{\text{s}}^{*}/\partial f$. In term of the covariance between *A* and $E_{\text{t}}^{*}$, the correlation coefficient is set to -1, the validity of which was confirmed in the fitting of Eq. 1. Assuming negligible errors in *γ* and *a*/*t*, the standard deviation *σ*_Efilm_ of $E_{\text{film}}^{*}$ is estimated by the error propagation on Eq. 2: $\sigma_{\text{Efilm}}^{2}=D_{\text{Es}}^{2}\sigma_{\text{Es}}^{2}+D_{\text{Esub}}^{2}\sigma_{\text{Esub}}^{2}$, where $D_{\text{Es}}=\partial E_{\text{film}}^{*}/\partial E_{\text{s}}^{*}$, $D_{\text{Esub}}=\partial E_{\text{film}}^{*}/\partial E_{\text{sub}}^{*}$, and *σ*_Esub_ is the standard deviation of $E_{\text{sub}}^{*}$.

## Results and Discussion

### Effective Young's Modulus of a Flat Tip and the Contact Radius

**Figure 3:**
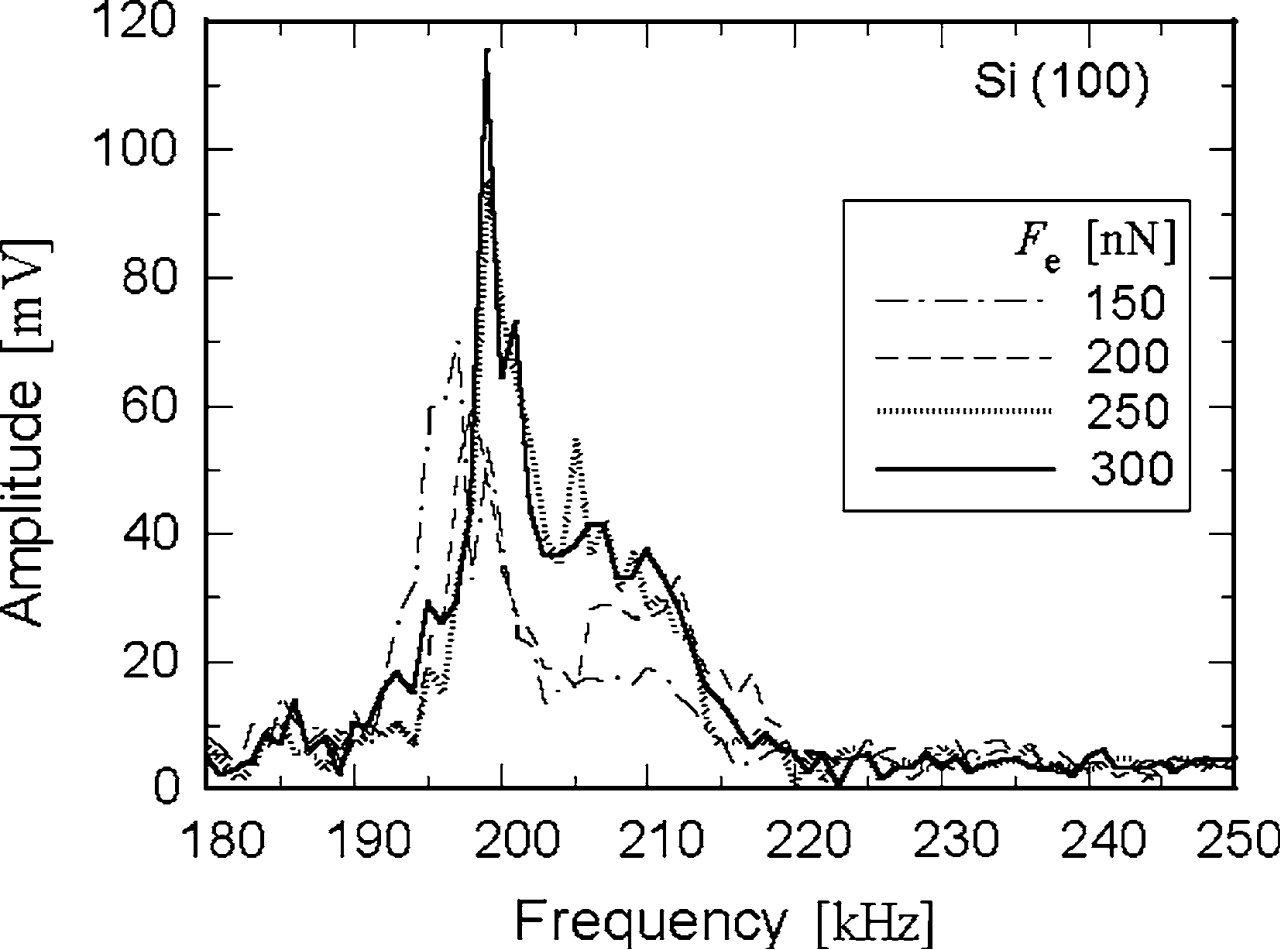
**The spectra of the CM cantilever vibration in contact with silicon (100) when increasing the contact force (*F*_e_)**.

The CM cantilever in free space measured *f*_0_ = 9.917 kHz for the fundamental resonant frequency. Figure 3 shows spectra for the reference, Si (100) wafer. The resonant frequency seems to become independent of the contact force (*F*_e_) when increasing *F*_e_. This reflects the constant contact area observed in the case of the flat tip.

**Figure 4:**
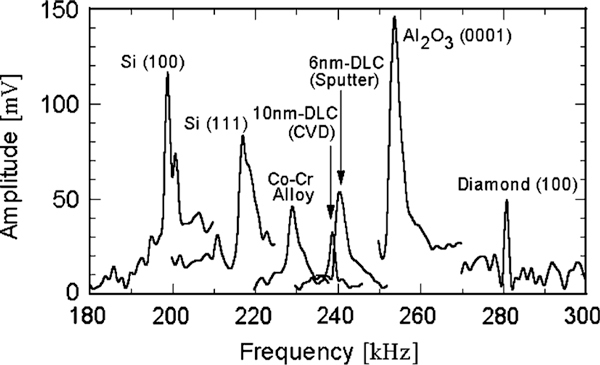
**The spectra for reference samples [Si (100), Si (111), Al_2_O_3_ (0001), and diamond (100)] and hard disk samples [6 nm-DLC (Sputter), 10 nm-DLC (CVD) and Co-Cr alloy (hard disk without DLC)]**.

To measure *f*, we set *F*_e_ to be a value as small as possible, at which the resonant peak was clear and settled in frequency. The value depended on the sample material. The resonance frequencies for Si (100), Si (111), Al_2_O_3_ (0001), and diamond (100) were *f* = 199.3 ± 1.3 kHz, 218.6 ± 1.9 kHz, 254.5 ± 1.1 kHz, and 281.0 ± 1.1 kHz, where *F*_e_ is set to 300, 400, 500, and 700 nN, respectively. The errors show the 95% confidence regions (±2*σ*). The excellent reproducibility was attained in the measurements for 5–10 different positions on each reference surface. Figure 4 shows examples of spectra for the reference samples.

**Figure 5:**
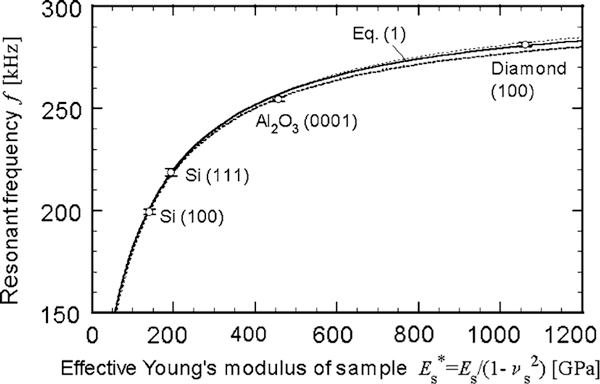
**The theoretical curve, which relates the resonant frequency to the effective Young's modulus of a sample, fitted to the experimental data (ο) for the reference**. The *error bars* and the broken curves indicate the 95% confidence regions, namely twice the standard deviations.

Fitting Eq. 1 to the relationship between the resonant frequencies measured for reference and the effective Young's moduli listed in Table 1, we determined $A(=af_{0}^{2}/k_{\text{c}})$ and $E_{\text{t}}^{*}$, which are hard to measure or estimate directly. Figure 5 shows the least-squares fit obtained for the reference samples, which yielded *A* = 0.2496 ± 0.0061 (± 2*σ*) m/kg and $E_{\text{t}}^{*}$ = 184.6 ± 8.8 (± 2*σ*) GPa. The errors for *A* and $E_{\text{t}}^{*}$ correlate, and the error's sign is taken opposite to each other.

Use of the values of *A*, *k*_c_, and *f*_0_ produced a reasonable contact radius *a* = 1.7 nm. Also, the value of $E_{\text{t}}^{*}$ is comparable to the averaged value for bulk platinum (196 GPa) and bulk titanium (129 GPa), but close to the value for platinum differently from the previous work ($E_{\text{t}}^{*}$ = 152.3 GPa) [12]. This would be on account of the contact area smaller than that in the previous work (*a* = 4.4 nm) [12]. In the contact deformation of the present tip, the contribution of surface layer (Pt) would dominate rather than the insert layer (Ti).

The square of the correlation coefficient (*r*^2^ = 0.9987) of the fit confirms the validity of the theory on a CM cantilever with a flat tip. The error bar for each data point and the broken curves in Figure 5 indicate the 95% confidence regions.

### Evaluation of DLC Thin Films

The samples coated with the 6-nm-thick DLC film (Sputter) and the 10-nm-thick DLC film (CVD) measured *f* = 240.4 ± 1.6 (± 2*σ*) kHz and *f* = 239.6 ± 0.5 (± 2*σ*) kHz, where *F*_e_ is set to 600 and 800 nN, respectively. The DLC coating shifted the resonant frequency to higher than that of the sample without DLC coating [*f* = 229.8 ± 1.6 (± 2*σ*) kHz (*F*_e_ = 500 nN)]. Also, the values of *f* for the two DLC films were alike despite the different thickness. This does not mean that the resonance is free from the substrate effects.

The effective Young's modulus of a sample was determined from the curve in Figure 5 to be $E_{\text{s}}^{*}$ = 310.5 ± 11.4 GPa, 305.2 ± 3.5 GPa, and 247.8 ± 8.2 GPa for the hard disks with 6-nm-thick DLC (Sputter), 10-nm-thick DLC (CVD), and without DLC coating, respectively. The errors are in the 95% confidence regions. The last one corresponds to $E_{\text{sub}}^{*}$. The value of $E_{\text{sub}}^{*}$ was similar to the modulus of Co-Cr alloys (230–280 GPa) [15,16].

Substituting the values of $E_{\text{sub}}^{*}$ and $E_{\text{s}}^{*}$ into Eq. 2, we obtained the effective Young's modulus of a film ($E_{\text{film}}^{*}$), where *γ* was calculated using *a* = 1.7 nm and *t* = 6 nm or 10 nm. The moduli were $E_{\text{film}}^{*}$ = 391.8 ± 34.7 (± 2*σ*) GPa and 345.1 ± 8.5 (± 2*σ*) GPa for the 6-nm DLC (Sputter) and the 10-nm DLC (CVD), respectively. The presence of substrate effects was clear in that the values of $E_{\text{s}}^{*}$ for the 6-nm-film-coated and 10-nm-film-coated samples were 20 and 10% less than the corresponding values of $E_{\text{film}}^{*}$, respectively. The values of $E_{\text{film}}^{*}$ were within the range of values reported for several DLC films, from 100 to 800 GPa [2-4]. Also, a good precision of 2*σ* < 10% was attained.

An error in *a*/*t*, which was neglected in the present evaluation, also causes uncertainty of the results. A postulated error of 20% in *a*/*t* results in a relatively small error of about 5 and 2.5% in $E_{\text{film}}^{*}$ for the DLC films of 6 nm thick (*a*/*t* = 0.283) and 10 nm thick (*a*/*t* = 0.17), respectively. The resulting error increases with *a*/*t*. Therefore, the contact radius (*a*) should be minimized.

The indentation depth *δ*_s_, namely the total displacement *δ* (= *F*_e_/k*) minus the tip deformation, can be estimated by taking account of the contribution of a sample, $k_{\text{s}}^{*}=2aE_{\text{s}}^{*}$, in the contact stiffness. The estimate was $\delta_{\text{s}}=F_{\text{e}}/k_{\text{s}}^{*}$ = 0.57 nm and 0.77 nm for the 6-nm-DLC and 10-nm-DLC samples, respectively. These indentation depths are 10% or less of the film thickness. The substrate effect should be carefully considered even when AFAM is applied. The present method provides the AFAM method of determining the elastic modulus for ultrathin films, eliminating the influence of a substrate. The sensitivity-enhanced AFAM proved to be sensitive enough for the determination of the ultrathin film elasticity and to have the excellent repeatability and reliability.

## Conclusion

The 6-nm-thick and 10-nm-thick diamond-like carbon (DLC) films on a hard disk were evaluated for the effective Young's modulus using sensitivity-enhanced atomic force acoustic microscopy. The modulus was determined from the measurements of the contact resonant frequency with the aid of a theory on indentation of a layered half-space. The moduli of the 6-nm DLC and 10-nm DLC were 391.8 ± 34.7 GPa and 345.1 ± 8.5 GPa, respectively, which reflected difference in the method of film deposition. The errors, the 95% confidence regions (± 2*σ*), show that this method gives a precise estimate of the effective Young's modulus.

## Appendix

Calculations of the modulus are alike for cubic crystals like diamond and silicon [12] and for trigonal crystals like sapphire (Al_2_O_3_). A trigonal crystal, however, has more constants, *c*_11_, *c*_12_, *c*_13_, *c*_14_, *c*_33_, and *c*_44_, than a cubic crystal. They relate stresses *σ*_*i*_ to strains *ε*_*i*_ (*i* = 1 - 6) as follows:

(4) $$\begin{array}{l} \sigma_{1}=c_{11}\varepsilon_{1}+c_{12}\varepsilon_{2}+c_{13}\varepsilon_{3}+c_{14}\varepsilon_{4}\\ \sigma_{2}=c_{12}\varepsilon_{1}+c_{11}\varepsilon_{2}+c_{13}\varepsilon_{3}-c_{14}\varepsilon_{4}\\ \sigma_{3}=c_{13}(\varepsilon_{1}+\varepsilon_{2})+c_{33}\varepsilon_{3}\\ \sigma_{4}=c_{14}(\varepsilon_{1}-\varepsilon_{2})+c_{44}\varepsilon_{4}\\ \sigma_{5}=c_{44}\varepsilon_{5}-c_{14}\varepsilon_{6}\\ \sigma_{6}=-c_{14}\varepsilon_{5}+(c_{11}-c_{12})\varepsilon_{6}/2 \end{array}$$

where the subscripts adopt an abbreviated notation (e.g., *σ*_1_ = *σ*_11_, and *σ*_4_ = *σ*_23_) [17]. All components are referred to Cartesian coordinates *x*_*i*_ (*i* = 1 - 3), where the *x*_1_ and *x*_3_ axes are taken along the a_1_-axis [1000] and the c-axis [0001], respectively. Calculation of the effective Young's modulus for the C-plane (0001) requires the Young's modulus *E*_[0001]_ in the direction of the c-axis. Eliminating *ε*_1_, *ε*_3_, and *ε*_4_ in Eq. 4 under the condition of uniaxial stressing *σ*_*i*_ = 0 (*i* ≠ 3), we can obtain the following formula from *E*_[0001]_ = *σ*_3_/*ε*_3_.

(5) $$E_{\left[0001\right]}=c_{33}-\frac{2c_{13}^{2}}{c_{11}+c_{12}}.$$

While the Poisson's ratio *ν*_[0001]_ depends on the direction in which lateral strain is measured, we let *ε*_1_ (= *ε*_2_) represent lateral strain for simplicity. This simplifies the calculation of *ν*_[0001]_:

(6) $$\nu_{\left[0001\right]}\approx -\frac{\varepsilon_{1}}{\varepsilon_{3}}=-\frac{\varepsilon_{2}}{\varepsilon_{3}}=\frac{c_{13}}{c_{11}+c_{12}}.$$

The elastic moduli for sapphire in Table 1 were obtained from Eqs. 5 and 6 together with the single-crystal constants, *c*_11_ = 490.2 GPa, *c*_12_ = 165.4 GPa, *c*_13_ = 113.0 GPa, and *c*_33_ = 490.2 GPa [18].
